# Deprivation scores and NHS practice payment trends in England 2018–2019 to 2023–2024:a multivariable analysis

**DOI:** 10.3399/BJGP.2025.0498

**Published:** 2026-05-06

**Authors:** Louis Steven Levene, Christopher Newby, George K Freeman, Emilie M Couchman, Richard H Baker

**Affiliations:** 1 Division of Public Health and Epidemiology, School of Medical Sciences, University of Leicester, Leicester, UK; 2 School of Medicine, University of Nottingham, Nottingham, UK; 3 Department of Primary Care and Public Health, Imperial College London, London, UK; 4 Division of Primary Care, Palliative Care and Public Health, University of Leeds, Leeds, UK; 5 Larkhill Medical Centre, Larkhill, Salisbury, UK

**Keywords:** chronic disease, healthcare costs, healthcare disparities, primary health care, social deprivation

## Abstract

**Background:**

Funding shortfalls persist for practices in the most deprived areas, despite capitation formula adjustments.

**Aim:**

To evaluate whether deprivation scores predicted practice payment trends between 2019 and 2024.

**Design and setting:**

Multivariable analysis was undertaken of English general practices (2018–2019 to 2023–2024), excluding practices with <750 patients or average payments >£500 per patient per year, using published data.

**Method:**

A quadratic mixed-effects model was fitted, using cluster-robust standard errors. The outcome was log-transformed average NHS practice payments per patient (net of deductions/reimbursements). The fixed effects were time (categorical), the Index of Multiple Deprivation (IMD) score (higher score indicates greater deprivation), and seven covariates (geographical, population, or organisational). The random effect was practices’ random intercepts.

**Results:**

Among 5726 included practices, median payments increased in nominal terms (8.6%) but decreased in real terms (–12.6% consumer price index [CPI] and –9.0% CPI for health). The IMD–payment trend relationship was curvilinear, peaking at IMD 49.8 (1.4% above mean deprivation, IMD 23.2), declining to 0.6% higher at IMD 70.0. More positive payment trends were associated with non-London regions, rurality, greater long-term conditions (LTCs) prevalence, and higher baseline payments; less positive trends were associated with more patients aged <16 years, larger lists, and personal medical services contracts. In interaction models, rurality increased whereas higher LTCs decreased IMD’s impact.

**Conclusion:**

Deprivation had a positive but diminishing association with payment trends as deprivation increased, moderated by geography and morbidity. Payment uplifts must match inflation. Funding formulas must better compensate for deprivation and morbidity, address the attenuated positive effect of deprivation in practices with more patients with LTCs, and minimise geographical inequalities.

## How this fits in

Because NHS practice payments have been criticised for ‘pro-rich inequity’, this study evaluated the association between deprivation scores and total payment trends in 2018– 2019 to 2023–2024. Inflation-adjusted total payments decreased while the deprivation–payment trend relationship was curvilinear, flattening as scores increased. These findings suggest persistent relative underfunding in the most deprived areas and geographical inequalities, particularly in urban areas and the London region. The mechanisms of these inequalities need further investigation, but remedies should include payment uplifts matching inflation, better alignment with direct deprivation measures, and weighting extended to other payment streams where appropriate.

## Introduction

General practices serving socioeconomically disadvantaged populations need additional resources, but have not always received them when compared with those serving more affluent populations.^1–9^ Since 2004, capitation payments directed to English general practices, accounting for about half of their NHS income,^10^ have been adjusted. As part of the NHS GP contract, the global sum allocation formula, also known as the Carr-Hill formula, weights capitation funding based on modelled estimates of practices’ patient workloads. These take into account two groups of factors, ‘drivers of workload’ and ‘unavoidable costs’^11,12^ (Supplementary Table S1). Carr-Hill originally used consultation length as a proxy for workload, which may be flawed as consultations are not necessarily longer in deprived areas.^13^ However, these areas are likely to experience higher morbidity that drives up healthcare ‘consumption’,^14^ and often suffer from uneven workforce distribution.^15^ Carr-Hill has only included proxy, never direct, measures of deprivation and morbidity,^16^ two important factors associated with health need. Even after age correction, the validity of Carr-Hill’s modelling for additional needs, using the measure’s standardised limited longstanding illness and the standardised mortality ratio for those aged <65 years, has been questioned.^11^

Associations have been found between higher total general practice payments per patient and greater life expectancy in England,^17^ and between higher capitation payments and better quality of primary care, as measured by Care Quality Commission ratings.^18^ In 2016, NHS England recognised that Carr-Hill had limitations and needed to be revised.^19^ A 2024 government review of the NHS acknowledged a *‘shortfall in funding for practices serving more deprived populations’*.^20^ At least one integrated care board has developed local general practice funding models to address inequities in Carr-Hill.^21^ In June 2025, the government pledged in its *Fit for the Future: 10 Year Health Plan for England* to strengthen care in the community and to *‘shift the pattern of health spending.’* ^22^ Achieving fairer funding will include a review of Carr-Hill; however, no details are currently available.

The context has evolved during and following the COVID-19 pandemic: total healthcare expenditures increased, although their *ad hoc* nature likely contributed to growing inefficiency,^23^ and there was a potential adverse impact on prevention and the management of long-term conditions (LTCs).^24^ Workforce reconfigurations have included substantial increases in non-doctor roles but not GPs,^25^ alongside increased triage, remote consulting,^26^ and growth in reported morbidity — the annual GP Patient Survey (GPPS) reported a post-pandemic increase in LTC prevalence of 19.6% (from 52.5% in 2021 to 62.8% in 2025).^27^ An updated investigation is needed into whether the previous suboptimal alignment of total NHS practice payments to deprivation in practice populations^4,5^ has improved or worsened since the pandemic. The study’s hypothesis was that practice payment trends continued to show little or no relative improvement in practices serving populations with the greatest deprivation; thus, the study’s research question was: how much did deprivation scores predict variations in the longitudinal trends of total NHS practice payments between 2018–2019 and 2023–2024, after adjusting for geographical, population, and organisational factors?

## Method

### Overview

This longitudinal, ecological multivariable analysis included practice-level data from published summary statistics (Supplementary Table S2). Organisation data service codes were used to define practices within each dataset before merging these.^28^ All included variables required consistently defined available data across the study period.

### Study population

The study population was English general practices from 2018–2019 to 2023–2024 fulfilling the following criteria: receiving NHS payments, classification as GP practices, and open for ≥1 full year. Practices with <750 patients or receiving very high adjusted average NHS payments (>£500 per registered patient per year) were excluded.

### Dependent variable

The outcome in each study year was average annual NHS payments per registered patient, from which deductions for pensions, levies, and prescription charge income, and reimbursements for premises payments and drugs were subtracted.^10^

### Independent variables

Where possible, the independent variables’ data were matched to each financial year. Unless otherwise stated, values were included for all 6 years from the continuous variables.

The central independent variable was the 2019 Index of Multiple Deprivation (IMD) score, an overall relative area-based measure of socioeconomic status that combines indicators from seven domains (income, employment, education, health, crime, barriers to housing and services, and living environment).^29^ Higher IMD scores indicate greater deprivation. The Office for Heath Improvement and Disparities publishes practices’ IMD scores:^30^ these were patient-weighted, calculated by NHS Digital by averaging the IMD of lower-layer super output areas according to the proportion of a practice’s patients resident in each. The current study used these scores, which reflect the deprivation profile of the practice’s catchment population, to assess their gradient relationship with payment trends.

Geographical, population, and organisational factors were adjusted for. For inclusion, a covariate needed to have a conceptually plausible relationship to either payments or deprivation, using a published population health research framework (Supplementary Figure S1),^31^ and to not be strongly correlated with others (coefficient <0.4) (Supplementary Figure S2). The latter was to reduce the risk of multicollinearity in the models. Some potential variables with strong correlations found in previous work using these datasets were eliminated.^32^ Seven independent variables fulfilled the criteria.

Two geographical categorical variables were included: the NHS commissioning region and rurality. England has seven NHS commissioning regions, responsible for the performance of all NHS organisations within their region (East of England, London, Midlands, North East and Yorkshire, North West, South East, and South West).^33^ London was the reference. The rural urban classification is an official statistical classification, used to distinguish urban (defined as settlements of ≥10 000 population) and rural (everything else) areas.^34^ Practices were classified as either rural or urban based on their postcode.^10^ Urban location was the reference.

Two continuous population variables were included: the percentages of practice patients aged <16 years^35^ and with LTCs from the GPPS^27^ to represent morbidity. The percentage aged <16 years^35^ was the only age band that did not correlate strongly with other variables. For morbidity, the authors of the current study considered using one of five potential Quality and Outcomes Framework disease registers,^36^ but all these correlated with at least one of the other independent variables, most commonly IMD. Also considered was using a self-reported broad ethnicity band from the GPPS,^27^ as defined in the England and Wales 2021 census,^37^ but this was moderately strongly correlated with the LTC variable (coefficient = 0.48). The GPPS data are weighted to adjust for response rates and practice population characteristics.

Three organisational variables were included: baseline payments (2018–2019), the practice list size (in 100s), and the type of NHS primary care contract at the start of the study period.^10^ Practices can have different contracts with the NHS, either general medical services (GMS), personal medical services (PMS), or alternative provider medical services (APMS). GMS was used as the reference. The first two variables were continuous and the latter categorical.

### Analyses

In data cleaning, implausible percentage values, >100% or negative, were replaced by missing values.

Descriptive statistics were used to assess continuous variables’ distributions. Very skewed distributions were log-transformed.

In the multivariable analyses, a linear mixed-effects regression model with practice random intercepts was fitted, including only practices with data in all 6 years. The initial model included time, IMD (linear term), and the seven covariates as fixed effects. Following diagnostic checks that suggested potential non-linearity in the deprivation–payment relationship, a quadratic IMD term was added, after mean-centring IMD, to address multicollinearity. Time was treated as categorical (factor) with six levels corresponding to each year (reference 2019), as model comparisons showed this specification provided a superior fit to continuous time (ΔAkaike information criterion [AIC] = –4961; likelihood-ratio test [LRT] χ²[4] = 234.5, *P*<0.001; Supplementary Table S3). The random effect was the practices' random intercepts, to account for unobserved baseline differences between them. To account for expected heteroscedasticity and clustering, standard errors clustered by practice with small-sample correction (CR2 type) were used to ensure valid inference. Additionally, whether the quadratic relationship differed across the payment distribution was examined by comparing practices in different payment quantiles.

Model diagnostics (residual plots, Cook’s distance) were assessed and robustness confirmed via sensitivity analyses that excluded influential observations (Cook’s distance ≥4/*n*).

Four supplementary models were fitted to check robustness and explore effect modification: a) testing interaction between IMD and rurality; b) testing interaction between IMD and LTC percentage; c) added percentage of patients of White ethnicity (with unreported values coded as 0%);^27^ and d) unadjusted (no subtractions) replaced adjusted payments. All these models treated time as categorical and used cluster-robust standard errors.

All models’ performances were assessed for multicollinearity, homogeneity of variance, and normality of residuals.

Statistical analyses were performed using R (version 4.5.1).

## Results

### Study population

There were 7279 practices registered for NHS payments in 2018–2019. Of these, 479 were transient, inactive, or providing a restricted range of services to very specific populations (such as walk-in clinics), leaving a potential study population of 6800 practices. A further 27 (0.4%) were excluded with <750 patients or with average payments >£500 per patient per year. Thus, 6773 practices were eligible. The models included 5726 practices (84.2% of those eligible) with data in all years (Supplementary Table S4).

### Descriptive statistics

Tables 1 and 2 show the descriptive statistics. The distributions of the payments and list size variables were highly skewed, requiring log-transformation for the model.

**Table 1.** Descriptive statistics of continuous variables (*N* = 5726 practices with data)

Variable	Practices, *n*	Mean	SD	Median	IQR	Skew	Kurtosis
**Adjusted total payments per patient**							
2019	5726	120.41	24.35	116.59	23.60	3.12	29.71
2020	5726	120.48	23.89	116.75	24.25	2.37	18.26
2021	5725	124.09	27.63	119.82	26.49	3.31	30.45
2022	5725	127.02	27.62	122.73	26.13	2.88	22.76
2023	5722	126.28	27.63	122.01	26.67	2.99	27.01
2024	5556	130.76	25.57	127.16	26.02	2.03	14.36
**IMD score 2019** ^**a**^	5726	23.18	11.67	21.13	16.43	0.78	3.13
**Aged <16 years, %**							
2019	5726	17.44	3.76	17.10	4.12	0.50	6.26
2020	5725	17.27	3.71	16.96	4.12	0.45	6.26
2021	5722	17.09	3.64	16.81	4.05	0.44	6.29
2022	5725	16.88	3.56	16.64	3.95	0.39	6.34
2023	5725	16.70	3.48	16.48	3.95	0.34	6.33
2024	5635	16.44	3.43	16.29	3.90	0.24	6.55
**With long-term condition, %**							
2019	5726	52.06	8.54	52.57	11.43	–0.29	3.23
2020	5725	53.05	8.79	53.52	11.79	–0.29	3.19
2021	5726	52.14	8.38	52.70	11.47	–0.25	2.98
2022	5726	54.86	9.05	55.25	12.28	–0.21	2.97
2023	5726	55.93	8.90	56.26	12.18	–0.20	2.89
2024	5644	60.48	8.86	60.95	12.05	–0.33	3.02
**Registered patients (100s)**							
2019	5726	87.26	52.31	78.05	60.55	2.53	19.08
2020	5725	88.60	53.08	79.00	60.93	2.55	19.47
2021	5722	89.26	53.47	79.53	61.12	2.53	18.92
2022	5725	90.81	54.32	80.59	61.66	2.49	18.25
2023	5725	92.34	55.53	81.83	62.45	2.48	17.61
2024	5628	95.17	57.57	84.24	63.49	2.49	16.76

^a^Higher IMD score indicates greater deprivation. IMD = Index of Multiple Deprivation. IQR = interquartile range. SD = standard deviation.

**Table 2.** Descriptive statistics of documented categorical variables (*N* = 5726)

Variable	*n*	%
**NHS commissioning region**		
London	1059	18.49
South West	375	6.55
South East	729	12.73
Midlands	1176	20.54
East of England	589	10.29
North West	891	15.56
North East and Yorkshire	907	15.84
**Rurality**		
Urban	4728	82.57
Rural	966	16.87
Missing	32	0.56
**NHS contract**		
GMS	4175	72.91
PMS	1449	25.31
APMS	102	1.78

APMS = alternative provider medical services. GMS = general medical services.

PMS = personal medical services.

Between 2018–2019 and 2023–2024 the median of average unadjusted payments per patient increased by 8.6% (£117.00 to £127.00) (Table 3). When practices were ranked by IMD score in deciles, the nominal increases in the medians of total payments were larger in the three least deprived deciles than in the three most deprived deciles, and, between 2021–2022 and 2022–2023, the medians decreased nominally in six of the seven most deprived deciles (Supplementary Figure S3). However, when adjusted for inflation between 2019 and 2024, the median decreased in real terms by –12.6% to £102.30 (using the consumer price index [CPI])^38^ and by –9.0% to £106.47 (using the CPI for health) (Table 3).^39^

**Table 3.** Median total payments per patient 2018–2019 to 2023–2024, nominal and inflation-adjusted (both general and health related)

Financial year	Median unadjusted (nominal) payment, £	CPI (2019 = £100), £	Median CPI-adjusted payment,^a^ £	CPIH (Oct 2018 = 100), £	Median CPIH-adjusted payment,^a^ £
**2018–2019**	117.00	100.00	N/A	100.00	N/A
**2019–2020**	117.00	100.85	116.01	102.50	114.15
**2020–2021**	120.00	103.46	115.99	104.73	114.58
**2021–2022**	123.00	112.87	108.97	105.93	116.11
**2022–2023**	122.00	121.08	100.76	110.47	110.44
**2023–2024**	127.00	124.15	102.30	119.28	106.47

^a^Adjusted payment (in 2019–2020 terms) = unadjusted payment × (100/[100 + CPI or CPIH increase]). CPI = consumer price index**. **CPIH = consumer price index for health. N/A = not applicable. Oct = October.

Between 2021 and 2024 the mean LTC prevalence increased by 16.00% (52.14% to 60.48%), having been previously stable (Table 1). The percentage of patients aged <16 years declined slightly from 2019–2024 (mean 17.44%–16.44%, standard deviation 3.76%–3.43%). The distribution was clustered, with very few adult-only (0.14%–0.40%) or paediatric-only (0%–0.01%) practices (Supplementary Table S5).

### Multivariable analyses

**Figure 1.**
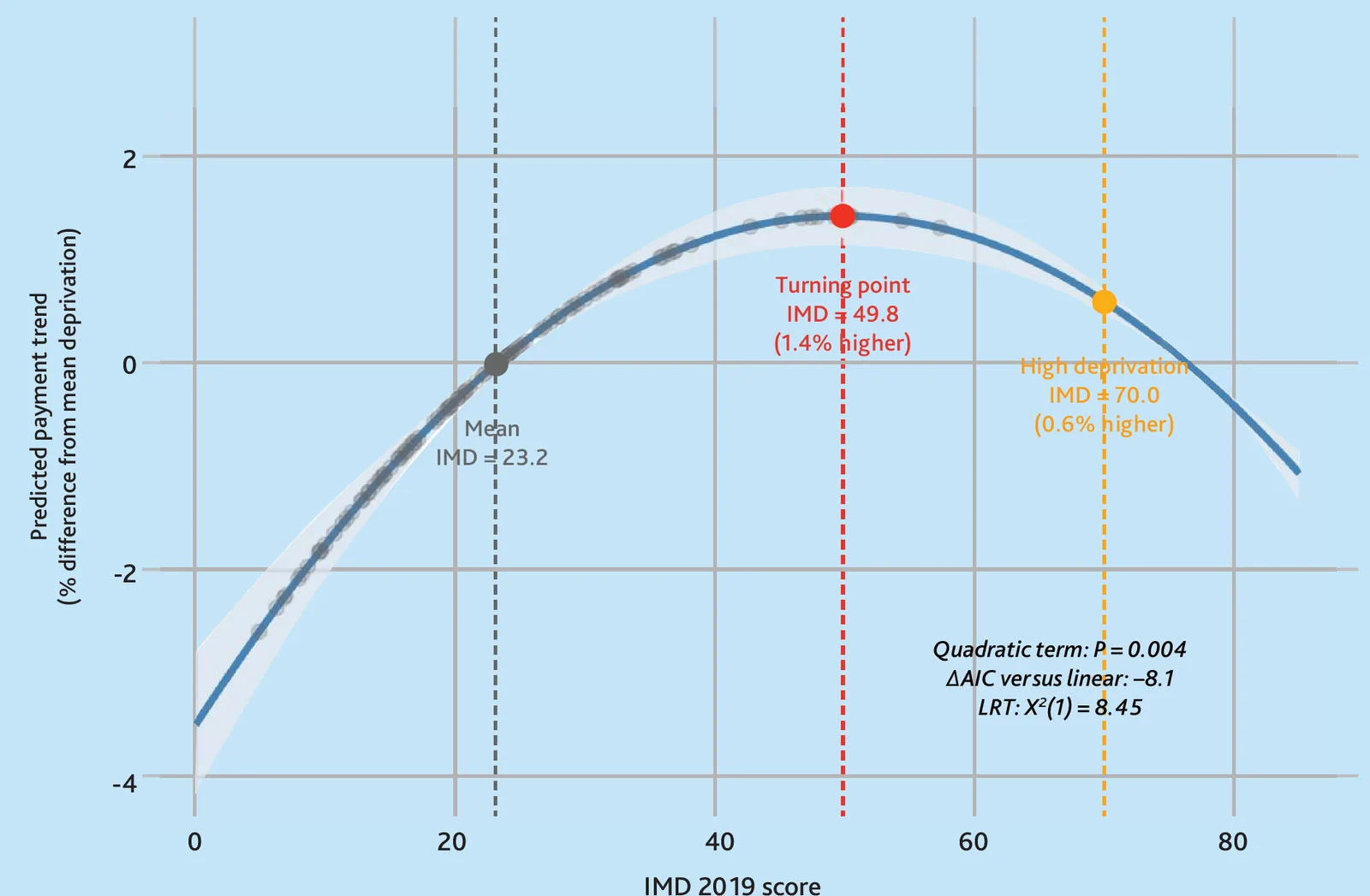
Curvilinear relationship between IMD and practice payment trends 2018–2019 to 2023–2024 Higher IMD score indicates greater deprivation. Predictions from quadratic mixed-effects model (notional *R* ^2^ = 0.730). Grey points represent sampled actual data, illustrating variation around the quadratic trend. Shaded area indicates approximate 95% CI. Further model comparison details are shown in Supplementary Table S3. AIC = Akaike information criterion. IMD = Index of Multiple Deprivation.

Table 4 presents the final mixed-effects model of practice payment trends (2018–2019 to 2023–2024). Initial model diagnostics suggested non-linearity; adding a quadratic term for IMD improved model fit (χ²[1] = 8.45, *P* = 0.004) (Figure 1). Both linear (*β* = 0.0011, 95% confidence interval [CI] = 0.0007 to 0.0014, *P*<0.001) and quadratic (β = −0.0000 × 10⁻⁵, 95% CI = −0.0000 × 10⁻⁵ to −0.0000 × 10⁻⁶, *P* = 0.003) IMD terms were significant, indicating diminishing positive payment trends with higher IMD scores. The relationship peaked at IMD 49.8 (1.4% above mean deprivation, mean IMD 23.2), declining to 0.6% higher at IMD 70.0.

**Table 4.** Linear mixed-model fit by restricted maximum likelihood of NHS practice payments 2018–2019 to 2023–2024 with IMD centred and quadratic IMD term^a^

**Variable**	*β*	95% CI	**SE**	***t*-test**	**df (Satt**)	***P*-value (Satt**)
**Fixed effects**						
(Intercept)	1.2900	1.0640 to 1.5120	0.1140	11.32	355.4	<0.001
2020 (versus 2019)	–0.0005	–0.0030 to 0.0020	0.0013	–0.38	5716.9	0.708
2021 (versus 2019)	0.0260	0.0225 to 0.0294	0.0017	14.89	5721.7	<0.001
2022 (versus 2019)	0.0469	0.0429 to 0.0509	0.0021	22.85	5658.9	<0.001
2023 (versus 2019)	0.0392	0.0347 to 0.0438	0.0023	17.04	5592.9	<0.001
2024 (versus 2019)	0.0734	0.0686 to 0.0783	0.0025	29.62	5186.2	<0.001
IMD 2019 (centred) (linear)	0.0011	0.0007 to 0.0014	0.0002	6.08	2082.7	<0.001
IMD 2019 sq (centred) (quadratic)	–0.0000	–0.0000 to –0.0000	0.0000	–3.05	536.9	0.003
Baseline log payments	0.7310	0.6869 to 0.7767	0.0228	32.06	344.7	<0.001
South West (versus London)	0.0360	0.0257 to 0.0464	0.0053	6.85	756.4	<0.001
South East (versus London)	0.0110	0.0030 to 0.0191	0.0041	2.68	1690.3	0.007
Midlands (versus London)	0.0143	0.0065 to 0.0222	0.0040	3.57	2329.4	0.000
East of England (versus London)	0.0253	0.0148 to 0.0358	0.0054	4.73	1377.8	<0.001
North West (versus London)	0.0281	0.0193 to 0.0369	0.0045	6.26	1883.9	<0.001
North East and Yorkshire (versus London)	0.0314	0.0228 to 0.0401	0.0044	7.16	2109.3	<0.001
Rural (versus urban)	0.0519	0.0422 to 0.0615	0.0049	10.53	1625.6	<0.001
Rurality missing (versus urban)	0.0035	–0.0214 to 0.0284	0.0122	0.29	31.6	0.776
Per cent aged <16 years	–0.0028	–0.0040 to –0.0016	0.0006	–4.54	737.9	<0.001
Per cent with LTC	0.0008	0.0005 to 0.0009	0.0001	7.51	3952.3	<0.001
List size, 100s	–0.0001	–0.0002 to –0.0001	0.0000	–4.81	290.0	<0.001
Contract PMS (versus GMS)	–0.0061	–0.0123 to 0.0000	0.0031	–1.98	2293.0	0.048
Contract APMS (versus GMS)	–0.0394	–0.0790 to 0.0002	0.0200	–1.97	113.8	0.053
**Random effects**	**Value**					
Residual variance, σ²	0.01					
Practice variance, τ₀₀	0.00					
Intraclass correlation	0.33					
**Model fit**						
Practices, *n*	5726					
Observations, *n*	34 172					
Marginal, *R*²	0.599					
Conditional, *R*²	0.730					

a Following detection of significant non-linearity (likelihood ratio test: χ²[1] = 8.45, *P *= 0.004), a quadratic IMD term was added. The negative quadratic coefficient (*β *= –1.96 × 10⁻⁵) indicates an ‘inverted U-shaped’ relationship, with payments peaking at IMD 50.2 (median deprivation level). All *P*-values based on cluster-robust standard errors (CR2 type, clustered by practice). Outcome was the log-transformed average adjusted total payments per patient per year. APMS = alternative provider medical services. df = degrees of freedom. IMD = Index of Multiple Deprivation. GMS = general medical services. PMS = personal medical services. Satt = Satterthwaite's method. SE = standard error. sq = squared.

Model-adjusted payment trends were non-linear, decreasing by 0.05% between 2019 and 2020 (*β* = –0.0005, *P* = 0.71) but increasing by 7.3% in between 2019 and 2024 (*β* = 0.0734, *P*<0.001). All regions had more positive trends than London (*β* = 0.011–0.036, all *P*<0.05), as did rural versus urban practices (*β* = 0.0519, *P*<0.001). Higher percentage of patients aged <16 years was associated with less positive trends (*β* = −0.0028, *P*<0.001); higher percentage LTC was associated with slightly more positive trends (*β* = 0.0008, *P*<0.001). Higher baseline payments were associated with more positive trends (*β* = 0.731, *P*<0.001). Larger list size was associated with slightly less positive trends (*β* = −0.0001, *P*<0.001). Compared with GMS contracts, PMS contracts were associated with a marginally significant less positive trend (*β* = −0.0061, *P* = 0.05), whereas the association with APMS did not reach significance (*β* = −0.0394, *P* = 0.053) (Table 4).

The model explained 73.0% of variance (conditional *R*²; intraclass correlation 0.33). Diagnostic checks indicated no concerning multicollinearity (all generalised variance inflation factors <2.1; Supplementary Table S6). Residual plots showed acceptable, although not perfect, homogeneity of variance and normality (Supplementary Figure S4).

Sensitivity analyses excluding influential observations (Cook’s distance ≥4/*n* and ≥5/*n* thresholds), as well as excluding one particularly influential practice, produced nearly identical coefficient estimates for the key IMD terms (linear: 0.00098–0.00108; quadratic: consistently −0.000021; Supplementary Table S7). To verify that a minor wording change in the 2024 GPPS LTC question did not affect the findings, a sensitivity analysis was conducted treating the 2024 LTC data as missing and refitted the main model. The coefficients for the deprivation terms (IMD linear and quadratic) were identical to six decimal places, and the model fit (AIC) was unchanged (Supplementary Table S8), confirming the robustness of the primary results.

### Supplementary analyses

High-payment practices were disproportionately rural (54% versus 14%) with smaller lists (*n* = 7540 versus *n* = 9550) (Supplementary Table S9). Stratified analyses showed different IMD–payment relationships as payment levels changed: slightly more positive trends for low-payment practices (*β* = 0.00035) but slightly less positive trends for high-payment practices (*β* = –0.00029), with no significant curvature within subgroups (both quadratic *P*>0.13). The full sample’s curvilinear pattern therefore appears to reflect compositional differences across the payment distribution (Supplementary Figure S5). Thus, the overall quadratic pattern may have resulted from combining groups with different baseline characteristics, consistent with the rurality–IMD interactions.

Four supplementary models tested robustness and identified modifiers of the deprivation–payment relationship (Supplementary Table S10). The positive IMD effect was stronger in rural than urban practices (quadratic interaction *P*<0.001) (Supplementary Figure S6). It was weaker in practices with higher LTC prevalence (linear interaction *P* = 0.004) (Supplementary Figure S7). Adding percentage White ethnicity revealed that practices with higher percentage White ethnicity had slightly more positive payment trends (*β* = 0.0003, *P*<0.001), although this did not substantially alter the IMD coefficients (Supplementary Table S10). Using unadjusted (no subtractions) payments per patient instead of adjusted payments per patient substantially weakened the association between IMD and payment trends, with the linear effect becoming non-significant (*P* = 0.101) and the quadratic effect attenuated (*P* = 0.024).

## Discussion

### Summary

In answer to the primary research question, statistically, higher deprivation (IMD) scores had a curvilinear association with payment trends between 2018–2019 and 2023–2024; the positive association lessened as deprivation increased. This finding was robust across sensitivity checks; however, during the study period real-terms payments decreased substantially after inflation adjustment (–12.6% retail prices and –9.0% health inflation). Additionally, the model found:

- statistically significant geographical disparities independent of deprivation scores, with less positive trends in urban practices and in the London region;
- more positive payment trends in practices with higher baseline payments; and
- less positive payment trends in practices with a younger age structure.

Supplementary analyses show that the IMD’s effect on payment trends was stronger in rural practices but weaker as the prevalence of LTCs increased, and less positive payment trends in practices with a greater ethnic minority population.

### Strengths and limitations

This study has a number of strengths. The model included 84.2% of English practices. Most characteristics showed small standardised mean differences (SMD <0.2) between included and excluded practices (Supplementary Table S11). Regional distribution differed most (SMD 0.40), with higher South West exclusion. However, similar IMD scores and payment levels suggest minimal bias for the curvilinear IMD–payment trend relationship. A robust process was used for selecting independent variables. Total payments was a broader outcome than specific income streams. Multivariable analysis provided a realistic approach for examining payment complexities. The model accounted for practice-level clustering and explained a substantial proportion of the outcome variance (high conditional *R*²), with diagnostic checks confirming appropriate fit (normally distributed residuals). The supplementary models helped to better understand the main model. The current study updated the evidence, examining payment trends across the pandemic.

Potential limitations included missing data for some variables (beyond the authors’ control). The direct NHS practice payments data do not include other indirect streams or private income, nor do they take account of community and outreach services. Although IMD is a standard research tool and has been used in funding analysis,^40^ it is a composite variable that could mask some heterogeneity even in very small areas.^41^ Its inclusion of a health domain (13.5%) could introduce potential confounding, and it does not capture short-term temporal changes. The GPPS addresses low-response rates (usually <30%) by weighting sampling and results to align with practices’ population characteristics, such as age and ethnicity.^27^ Cluster-robust methods provided reliable *P*-values and confidence intervals despite heteroscedasticity (expected in large observational data) and clustering, although they may reduce power. Sensitivity analyses excluding influential observations did not alter coefficient estimates or model fit statistics (Supplementary Table S7). Ecological studies find associations but cannot prove causality, including exclusion of potential reverse causality between deprivation and payments.

### Comparison with existing literature

Although providing updated evidence, the current findings aligned with the authors’ previous pre-pandemic analyses, showing that increased deprivation was weakly associated with higher practice payments, both cross-sectionally^4^ and longitudinally,^5^ and with other studies showing that, overall, practices serving more deprived populations had received little or insubstantial additional payments.^6–8^ Another study assessed *‘inequity in payments’* to English primary care providers from 2014–2022, comparing multiple definitions of need, and found *‘pro-rich inequity in total payments for most definitions of need’*.^9^ More deprived primary care networks attained fewer performance-related investment and impact fund points in 2022–2023, hence less additional funding.^42^ The above evidence suggests current payment systems remain insufficiently responsive to increases in diverse measures of either deprivation or need, a situation unchanged post-pandemic.

### Implications for research and practice

The study findings have clear practical significance:

- the inflation-adjusted decrease in NHS general practice payments, since before the pandemic, will not help practices trying to manage rising workloads from increased LTC prevalence and from the *10 Year Health Plan for England*’s proposal to shift care from hospitals into the community;^22^
- the funding formula seems to inadequately address relative underfunding, particularly for practices serving the most deprived areas, although this study’s observational design precludes any causal inferences;
- independent of deprivation, the current analysis indicates possible geographical inequalities, particularly in urban areas and the London region, with further investigation needed to better understand the mechanisms; and
- the attenuated positive association between deprivation and payment trends in practices with high LTC prevalence could, if unaddressed, risk worsening inequities and the persistence of the inverse care law.^2^

Potential remedies could include:

- ensuring payment uplifts match inflation;
- thoroughly reviewing the Carr-Hill formula to better align with direct deprivation measures and reduce potential overcompensation; and
- examining other payment streams for extending weighting and for switching performance targets to more work-sensitive metrics, where appropriate.

These actions may help the *10 Year Health Plan for England* to achieve its pledge to close health inequalities.

## Data

All data and analytical code for this study are publicly available on Zenodo at: 10.5281/zenodo.19636272. The repository includes the raw dataset and the complete R script used for analysis, preserving all iterative steps and exploratory work.
